# Systems physiology of the baroreflex during orthostatic stress: from animals to humans

**DOI:** 10.3389/fphys.2014.00256

**Published:** 2014-07-08

**Authors:** Atsunori Kamiya, Toru Kawada, Masaru Sugimachi

**Affiliations:** Department of Cardiovascular Dynamics, National Cerebral and Cardiovascular Center Research InstituteSuita, Japan

**Keywords:** baroreflexes, systems analysis, sympathetic nerve activity, autonomic nervous system, integrative physiology

## Abstract

The baroreflex is a key mechanism involved in the control of arterial pressure (AP) during orthostasis in humans. However, the baroreflex is a closed-loop feedback system, from baroreceptor pressure input to systemic AP, and therefore requires open-loop experiments to identify its system characteristics. The requirement limits our ability to identify baroreflex system characteristics in humans. Open-loop research in animals has revealed dynamic and static characteristics of the two baroreflex subsystems: the neural and peripheral arcs. The neural arc, from baroreceptor pressure input to sympathetic nerve activity (SNA), has high-pass dynamic characteristics, indicating that more rapid change in input AP causes greater response in SNA. In contrast, the peripheral arc, from SNA input to systemic AP, has low-pass characteristics. Orthostasis increases the gain of the neural arc, which compensates for the lower transfer gain of the peripheral arc and in turn maintains total baroreflex function. Here, I discuss the possibility that baroreflex subsystem characteristics identified in animals can be applicable to the human sympathetic response to orthostasis, with a focus on loading speed-dependence of orthostatic sympathetic activation.

## Introduction

The maintenance of arterial pressure (AP) under orthostatic stress from gravitational fluid shift is of great importance in humans (Eckberg and Sleight, 1992) and animals (rats, rabbits etc.) that spend most of their time in a head-up posture and that frequently stand during their daily life. The baroreflex is a key mechanism involved in the control of AP during orthostasis since baroreflex failure leads to severe orthostatic hypotension (Cooke et al., 1999; Fu et al., 2009). The baroreflex is a negative-feedback closed-loop system, from baroreceptor pressure input to systemic AP, and therefore needs open-loop surgical operation to identify its system characteristics (Ikeda et al., 1996), which is fundamentally impossible in human research. Animal research that has used open-loop baroreflex and white-noise input techniques (system identification) have clarified dynamic and static transfer characteristics of the two baroreflex subsystems, the neural arc and peripheral arc, during orthostasis as described below.

Regarding the dynamic transfer characteristics, the neural arc, from baroreceptor pressure input to SNA, has high-pass dynamic characteristics, which means that a more rapid change in AP results in a greater response in SNA, whereas the peripheral arc, from SNA input to systemic AP, has low-pass dynamic characteristics (Kawada et al., 2002; Kamiya et al., 2005c). The open-loop transfer function of the neural arc is able to predict time-series SNA responses to drug-induced AP changes with an *r*^2^ of 0.9, whereas the closed-loop-spontaneous transfer function cannot with a negative *r*-value (the inverse of measured responses) (Kamiya et al., 2011). In addition, orthostatic stress, caused from movement from a horizontally supine position, increases the transfer function gain of the neural arc, which helps compensate for the lower transfer function gains of the peripheral arc during orthostasis. This in turn helps maintain total baroreflex function (Kamiya et al., 2008). Regarding the static transfer characteristics, orthostatic stress resets the neural arc (baroreceptor pressure-SNA curve) to a higher SNA level (in the kidney and the heart), which compensates for the reduced presser responses to an increase in SNA in the peripheral cardiovascular system and helps prevent postural hypotension (Kamiya et al., 2005b, 2010).

Although system identification of the baroreflex is a useful tool for understanding baroreflex function in a variety of physiological and pathophysiological conditions, it requires surgical operation to open the baroreflex loop. The requirement of an open-loop experimental condition limits its application in human research. Therefore, the system characteristics of the baroreflex identified in animals, particularly the dynamic transfer function characteristics, have not been related to human baroreflex physiology.

Here, I discuss the possibility that baroreflex subsystem characteristics identified in animals can be applicable to the human sympathetic response to orthostasis. I will focus on the high-pass filter dynamic transfer function characteristics identified in muscle, cardiac and renal SNA of anesthetized rabbits (Kamiya et al., 2005c). I will also discuss whether transfer function characteristics identified in animals can explain the previously reported finding in humans that slow head-up tilt causes lower activation of muscle SNA (MSNA): loading speed-dependence of orthostatic sympathetic activation in humans (Kamiya et al., 2009).

## Speed-dependence of orthostatic sympathetic activation in humans

**Figure 1:**
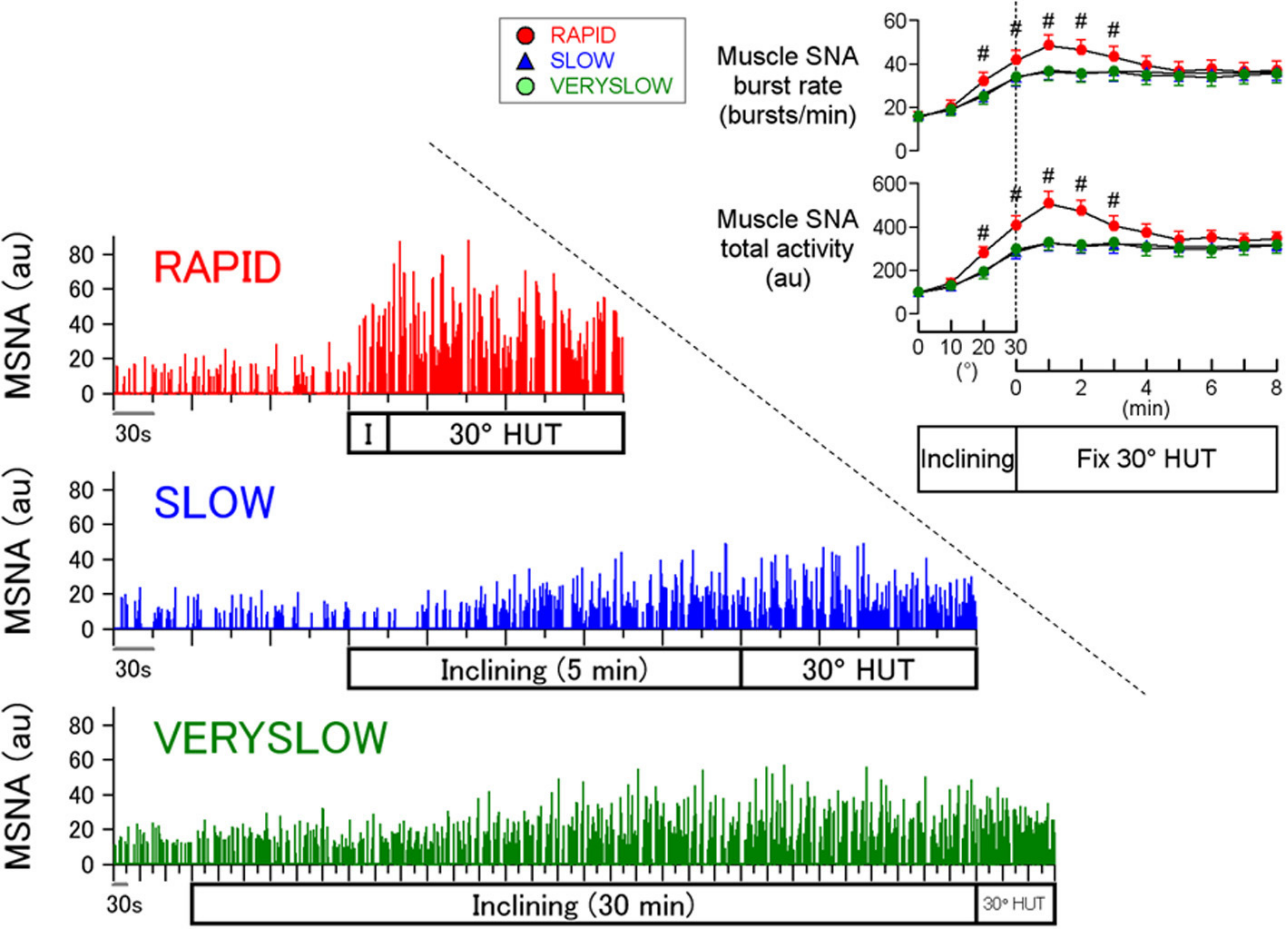
**Human MSNA responses to HUT tests with RAPID, SLOW, and VERYSLOW inclining speed.** ^#^*P* < 0.05 vs. SLOW and VERYSLOW tests. Error bars denote standard error. Modified from the study: Kamiya et al. (2009).

A stronger orthostatic stress causes greater MSNA response during head-up tilt (HUT) and thus it is well known that orthostatic MSNA activation is amplitude-dependent (Eckberg and Sleight, 1992; Cooke et al., 1999; Fu et al., 2009). In contrast, less attention has been paid to the effects of loading speed of orthostatic stress on orthostatic sympathetic activation in humans. Our previous study (Kamiya et al., 2009) examined whether the inclining speed of HUT influences the MSNA response to passive 30° HUT, independent of the magnitude of HUT, using inclining speeds of 1, 0.1, and 0.0167°/s (RAPID, SLOW, and VERYSLOW tests, respectively), in 12 healthy subjects (Figure 1). Calf MSNA (averaged over every 10° tilt angle) increased during inclination from 0 to 30°, with greater increases in the RAPID test than SLOW and VERYSLOW tests. In addition, only the RAPID test caused MSNA overshoot after reaching 30° HUT, whereas the SLOW and VERYSLOW tests did not. These results indicate that slower HUT results in smaller activation of MSNA suggesting that HUT-induced sympathetic activation depends partially on the speed of inclination during HUT in humans. The speed-dependence was also found in the high frequency amplitude of R-R interval variability (an index of cardiac vagal nerve activity), that decreased to a lesser extent during the inclination and after reaching 30° in the VERYSLOW test compared to the RAPID test.

## Characteristics of baroreflex subsystems in animals: neural and peripheral arcs

Previous system identification using open-loop experiments and transfer function analysis, commonly used in engineering, have revealed that in anesthetized animals (for example, rabbits), the transfer function of the neural arc (baroreceptor pressure to SNA) approximates derivative characteristics in the frequency range below 0.8 Hz, and high-cut characteristics of frequencies above 0.8 Hz (Kamiya et al., 2005c).Therefore, the neural arc transfer function (*H_N_*) can be modeled by using Equation A as follows:

(A) $$H_{N}(f)=-K_{N}\frac{1+\frac{f}{f_{c1}}j}{{\left(1+\frac{f}{f_{c2}}\right)}^{2}}\exp(\text{​}-2\pi fjL)$$

where *f* and *j* represent the frequency (in Hz) and imaginary units, respectively; *K_N_* is static gain (in a.u./mmHg); *fc*1 and *fc*2 (*fc*_1_ < *fc*_2_) are corner frequencies (in Hz) for derivative and high-cut characteristics, respectively; and *L* is pure delay (in seconds), that would represent the sum of delays in the synaptic transmission at the baroreflex central pathways and the sympathetic ganglion. The dynamic gain increases in the frequency range of *fc*_1_ to *fc*_2_, and decreases at frequencies above *fc*_2_.

In addition, the transfer function of the peripheral arc (SNA to systemic AP) approximates the second-order low-pass filter with a lag time in rabbits (Kamiya et al., 2005c). Therefore, the peripheral arc transfer function (*H_p_*) can be modeled by using Equation B as follows:

(B) $$H_{P}(f)=\frac{K_{P}}{1+2\zeta \frac{f}{f_{N}}j+{\left(\frac{f}{f_{N}}j\right)}^{2}}\exp(\text{​}-2\pi fjL)$$

where *K_P_* is static gain (in mmHg/a.u.); *f_N_* and ζ indicate natural frequency (in Hz) and damping ratio, respectively; and *L* is pure delay (in seconds), that would represent the sum of delays in the synaptic transmission at neuroeffector junction and the intracellular signal transduction in the effector organs.

## Numerical simulation of human MSNA response to hut using animal baroreflex characteristics

**Figure 2:**
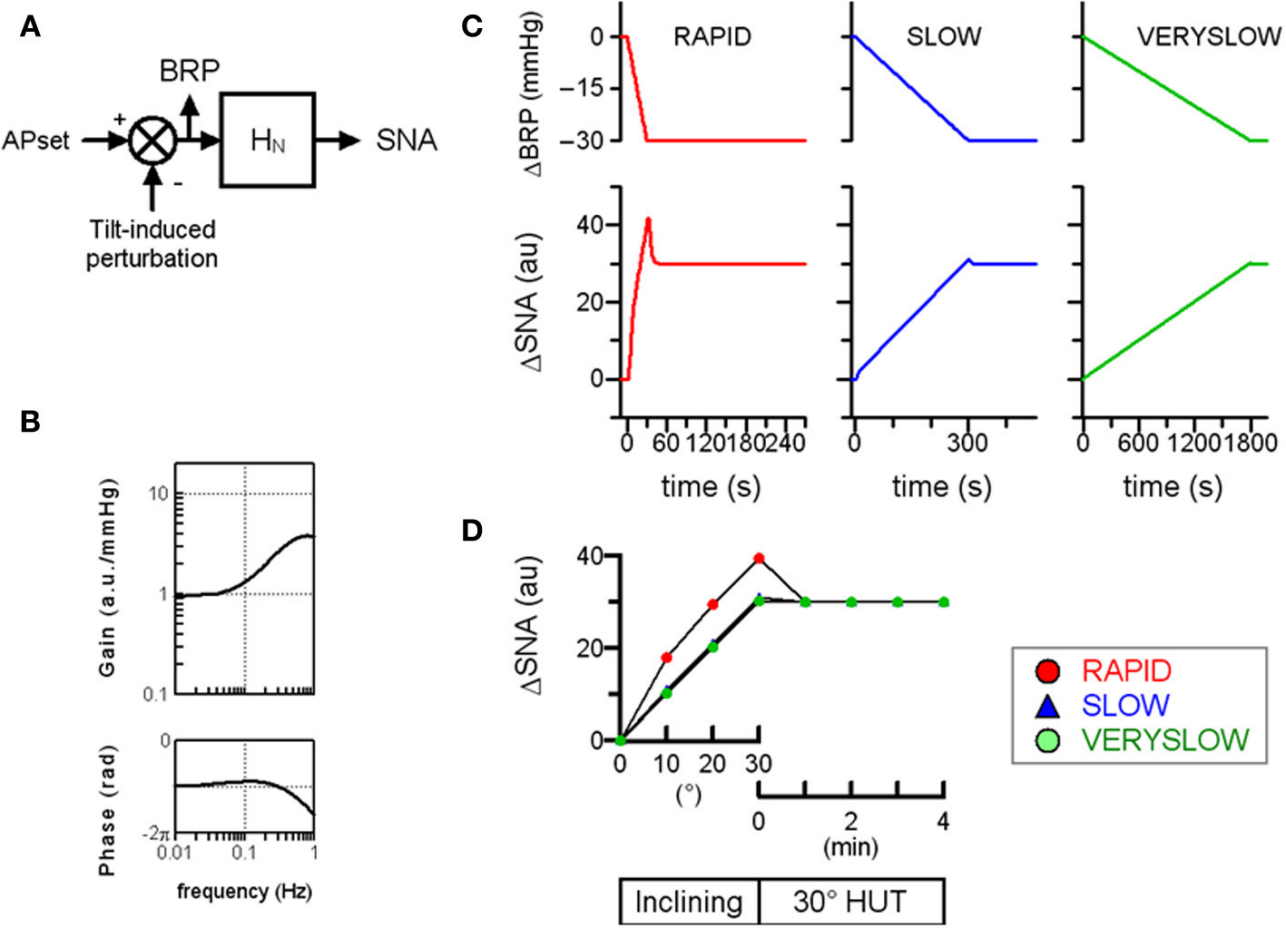
**Simulation of orthostatic activation of SNA in response to HUT at different inclining speeds in the baroreflex open-loop condition. (A)** A block diagram of baroreflex control of SNA containing the neural arc transfer function (*H_N_*). We assigned maximum tilt-induced perturbation of −30 mmHg in the simulations, equivalent to −1, −0.1, and −0.0167 mmHg/s in RAPID, SLOW and VERYSLOW HUT tests, respectively. We set APset at 100 mmHg. BRP; baroreceptor pressure. **(B)** The gain and phase function of *H_N_*. The gains *K_N_* are set at 1 au/mmHg. The *fc*_1_, *fc*_2_, and *L* are set at 0.1, 0.8, and 0.2, respectively, based on animal experimental data. **(C)** Simulated time series changes in BRP and SNA at conditions set in **(A,B)**. The RAPID test resulted in greater activation and an overshoot of SNA, compared with SLOW and VERYSLOW tests. **(D)** Simulated SNA data (same as **C**) of the RAPID (open circle), SLOW (closed triangle), and VERYSLOW (closed circle) HUT tests plotted on the same axes. Upper X-axis indicates that data are averaged over every 10° tilt angle during inclination from 0° supine to 30° HUT, while the lower X-axis indicates that data are averaged over every 1 min after reaching 30° HUT. Modified from the study: Kamiya et al. (2009).

First, a numerical simulation of the open-loop baroreflex condition was performed based on transfer functions actually identified in anesthetized animals (Figure 2). Since the increases in thoracic impedance averaged over tilt angle were similar in the RAPID, SLOW, and VERYSLOW HUT tests, the gravitational fluid shift directed toward the lower part of the body (such as the abdominal vascular bed and lower limbs) may be similar in all three tests at a tilt angle of 30°. Therefore, we assumed that the tilt-induced pressure perturbation was similar in the three HUT tests except for the speed. The numerical simulation indicates that in the open-loop baroreflex condition, the RAPID HUT test (1°/s) caused greater MSNA activation than SLOW (0.1°/s) and VERYSLOW (0.0167°/s) tests. This result appears to be consistent with our data observed in humans (Figure 1), and raises the possibility that the baroreflex control of SNA in humans also has high-pass filter characteristics.

**Figure 3:**
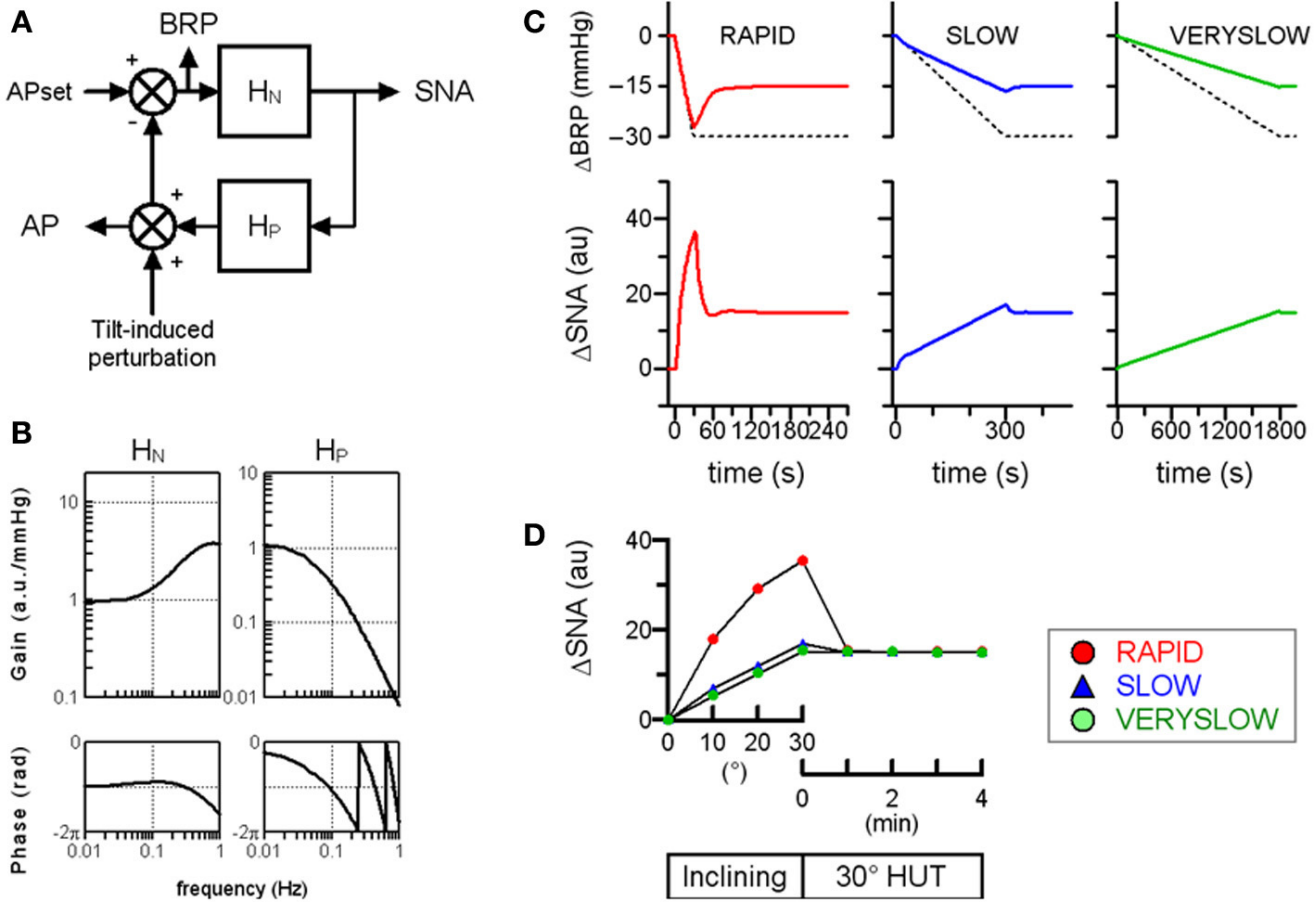
**Simulation of orthostatic activation of SNA in response to HUT at different inclining speeds in the baroreflex closed-loop condition. (A)** A block diagram of total arc baroreflex system that contains the neural arc transfer function (*H_N_*) and theperipheral arc transfer function (*H_P_*). We assigned maximum tilt-induced perturbation of −30 mmHg in the simulations, equivalent to −1, −0.1, and −0.0167 mmHg/s in RAPID, SLOW and VERYSLOW HUT tests, respectively. We set APset at 100 mmHg. BRP; baroreceptor pressure. **(B)** The gain and phase function of *H_N_* and *H_P_*. In the model of *H_N_*, the gain (K_*N*_) is set at 1 au/mmHg, while the remaining parameters are set similarly in Figure 2. In the model of *H_P_*, the *K_P_, f_N_*, ζ and *L* are set at 1, 0.07, 1.4, and 1, respectively, based on observation in animals. **(C)** Simulated time series changes in BRP (solid lines, upper panels) and SNA (lower panels) at conditions set in **(A)** and **(B)**. The dotted lines in upper panels indicate the tilt-induced perturbations. The RAPID test results in greater activation and an overshoot of SNA compared to SLOW and VERYSLOW tests. **(D)** Simulated SNA data (same as **C**) of the RAPID (open circle), SLOW (closed triangle), and VERYSLOW (closed circle) HUT tests plotted on the same axes. Upper X-axis indicates that data are averaged over every 10° tilt angle during inclination from 0° supine to 30° HUT, while the lower X-axis indicates that data are averaged over every 1 min after reaching 30° HUT.

Next, the relevance of baroreflex control of SNA to the speed-dependence in orthostatic MSNA activation was also confirmed by performing a numerical simulation mimicking the closed-loop baroreflex condition (Figure 3). The neural arc is arranged in series with the peripheral arc. Therefore, the total baroreflex loop is a negative-feedback control system that senses AP as baroreceptor pressure and regulates systemic AP. The simulation data indicated that the RAPID HUT test caused greater MSNA activation than the slower HUT tests, which is partially consistent with our previously observed data obtained in humans.

However, the dynamic transfer function characteristics of the neural arc cannot explain the 3-min overshoot of MSNA activation after reaching 30° HUT posture in the RAPID HUT test. In the numerical simulations, MSNA overshoot lasts less than 20 s in both baroreflex open-loop and closed-loop conditions. Accordingly, other mechanisms may be responsible for the overshoot of the orthostatic MSNA response in the faster HUT test. One possibility is a vestibulo-sympathetic response (Hammam et al., 2014; Yates et al., 2014). Another possibility is an effect of antigravity muscle contraction on SNA, since head-up suspension that removes antigravity muscle contractions caused smaller MSNA activation than HUT (Shamsuzzaman et al., 1998). Interestingly, without the numerical simulations based upon actual open-loop system identification in animals, it is difficult to predict the length of MSNA overshoot mediated by the baroreflex and the potential involvement of mechanisms other than the baroreflex.

## Baroreflex dynamic transfer characteristics and basic classic data of baroreceptor afferent

The baroreflex dynamic transfer function identified by a white-noise and open-loop method (Kamiya et al., 2011) is a transfer characteristics from baroreceptor pressure input to SNA in the baroreflex neural arc and that from SNA input to systemic AP in the baroreflex peripheral arc. The dynamic transfer function shows a linear component of the system, and is able to predict a time-series SNA response to randomly drug-induced (phenylephrine and nitroprusside infusions) AP changes in closed-loop condition with a high degree-of accuracy (*r*^2^ of 0.9 ± 0.1) (Kamiya et al., 2011). However, the baroreflex dynamic transfer function is limited to address basic classic data of baroreceptor afferent, in particular the contrasting effects of static and pulsatile pressure on carotid baroreceptor activity in dogs (Chapleau and Abboud, 1987). For example, although the single unit baroreceptor afferent nerve activity increased in response to an increase in baroreceptor pressure, the pulsatile baroreceptor pressure resulted in lower threshold as compared with static (ramp-like) baroreceptor pressure. Another example is a baroreceptor afferent response to a shift from static to pulsatile pressure. A pulsatility increased afferent nerve activity at low mean arterial pressures, whereas it decreased afferent nerve activity at high mean arterial pressures. These interesting observations may relate with a non-linear component of baroreflex system.

## Conclusion

System identification can be a powerful tool in the research of complex biosystems. However, its application for human research is often difficult since it requires open-loop surgical operation when the system is a closed-loop biosystem, which applies to the baroreflex. As a helpful challenge, I show the possibility that system identification based analysis and numerical simulation using baroreflex subsystem characteristics identified in animals can contribute to our understanding of human sympathetic physiology under orthostasis (Kamiya et al., 2005a, 2009).

### Conflict of interest statement

The authors declare that the research was conducted in the absence of any commercial or financial relationships that could be construed as a potential conflict of interest.
